# Polymerization-Induced Electrostatic Self-Assembly Enables Noncytotoxic Polyplex Formation for Gene Delivery

**DOI:** 10.1021/acsmaterialslett.6c00077

**Published:** 2026-04-15

**Authors:** Maria A. Castillo, Jungyeon Kim, Francesca Patel-Burrows, Xueyuan Li, Antony Adamson, Lee A. Fielding, Samuel T. Jones

**Affiliations:** † Department of Materials, 5292 University of Manchester, Manchester M13 9PL, United Kingdom; ‡ Henry Royce Institute, 5292 University of Manchester, Manchester M13 9PL, United Kingdom; ¶ Genome Editing Unit, 5292 University of Manchester, Manchester M13 9PL, United Kingdom; § School of Chemistry, 1724 University of Birmingham, Edgbaston B15 2TT, United Kingdom

## Abstract

Gene delivery is a cornerstone of both basic research and gene therapy, where it holds the key to treating genetic disorders at their source. However, it does not adhere to one-size-fits-all solutions. Cargo-tailored and time-saving approaches are urgently needed for different applications. Photoinduced electron/energy transfer reversible addition-fragmentation chain-transfer (PET-RAFT) polymerization is a technique that enables the synthesis of a wide range of well-defined polymers, allowing for rapid reaction condition screening in small volumes. In this work, polymerization-induced electrostatic self-assembly (PIESA) based on PET-RAFT polymerization is demonstrated to facilitate the simultaneous synthesis and assembly of pDNA polyplexes for transfection. The as-prepared polyplexes are shown to be less cytotoxic than their conventionally assembled counterparts and act as more effective gene delivery vehicles in transfection trials. This strategy provides a versatile platform that facilitates rapid screening of cell-compatible copolymer nanoparticle compositions for gene delivery in ultralow volumes.

## Graphical abstract

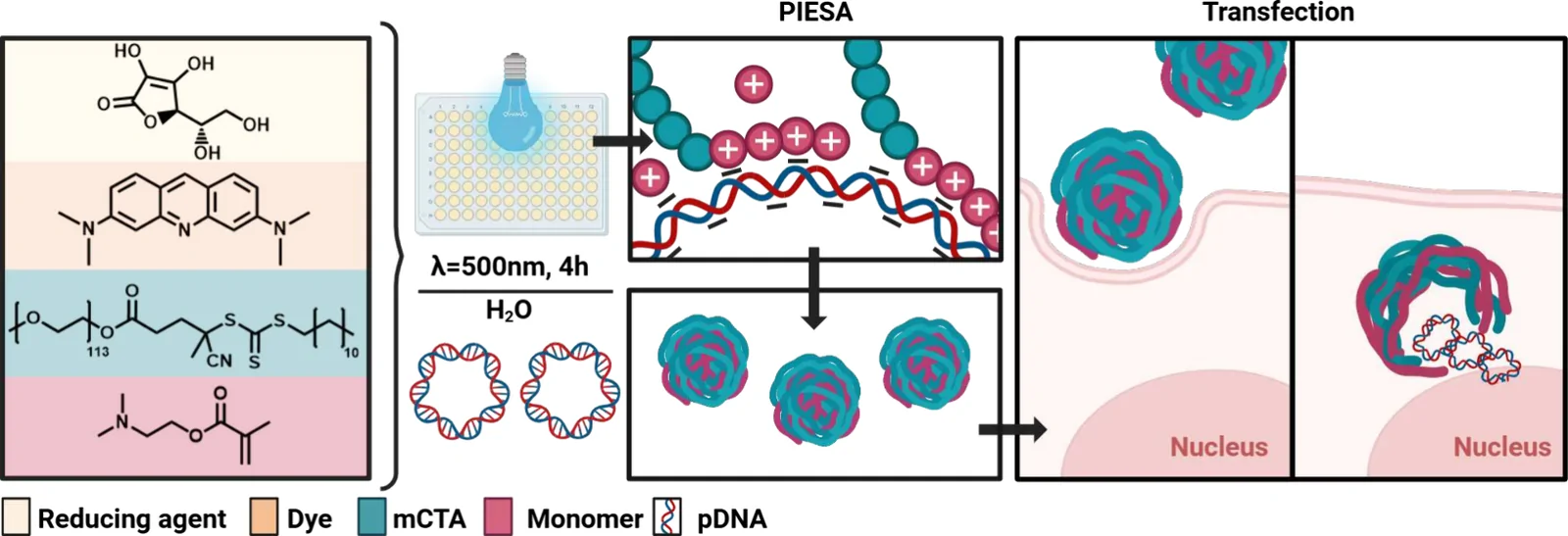

Transfection, the introduction of exogenous genes into cells, is a fundamental technique in molecular biology and biotechnology. It enables the study of gene expression^1^ and the production of recombinant biomolecules^2^ such as antibodies, enzymes, and peptides.^3^ It also serves as a nonviral alternative in vaccine development and gene therapy, where polymeric carriers and lipid nanoparticles avoid safety concerns associated with viral vectors.^4–6^ DNA delivery is inherently challenging due to the negatively charged cell membrane, which prevents spontaneous uptake.^1,7,8^ Nanovehicles are therefore required to overcome barriers including stability, cellular uptake, endosomal escape, and toxicity.^1,7^ Lipid nanoparticles are widely used but can suffer from cargo-size limitations, toxicity at high concentrations, and extracellular instability, affecting handling and in vivo performance.^9–11^

In contrast, cationic polymers offer high tunability through straightforward chemical modification.^12^ Their structure can be tailored to improve stability and biocompatibility through the incorporation of molecules such as polyethylene glycol (PEG) or poly(lactic-*co*-glycolic acid) (PLGA).^13–15^ Several techniques can be used for the synthesis of polymers for transfection. One such technique is reversible addition-fragmentation chain-transfer (RAFT) polymerization, which enables the synthesis of polymers with controlled molecular weights and low molar mass dispersities, and is compatible with PEG-containingblock copolymer formation using macromolecular chain-transfer agents (mCTAs).^16–18^ However, traditional thermally-initiated, RAFT polymerisation requires oxygen-free conditions.^19^ Photoinduced electron/energy transfer RAFT (PET-RAFT) overcomes this limitation by enabling polymerization in the presence of oxygen at low volumes and in aqueous media through light-mediated radical generation.^20–22^

Advanced gene delivery systems require precise control over the polymer architecture to tune properties such as stability and cellular uptake.^23^ RAFT and PET-RAFT can generate a wide range of polymer architectures, including block copolymers and higher-order structures.^21^ Polymerization-induced self-assembly (PISA) enables the formation of nanoparticles with defined morphologies during polymerization.^24–26^ Similarly, polymerization-induced electrostatic self-assembly (PIESA) uses electrostatic interactions between growing polymers and charged templates.^27^ Previous work has demonstrated PIESA using polyelectrolytes and siRNA templates; however, its application to gene delivery-relevant nucleic acids remains unexplored.^27–30^ To accelerate the development of gene delivery polymers, more efficient screening strategies are required.^22,31^ We propose that combining the oxygen-tolerant PET-RAFT approach with PIESA in the presence of DNA as a template should allow for the simultaneous synthesis and assembly of polyplexes. This should give precise control over particle characteristics and address current transfection challenges such as toxicity and efficiency. Herein, a strategy to achieve “one-pot” PIESA to readily generate polyplexes for transfection is demonstrated.

PEG–poly(2-(di­methyl­amino)­ethyl methacrylate) (PEG–PDMAEMA) block copolymers have previously been shown to assemble into polyplexes for transfection due to ionic interactions between the cationic PDMAEMA block and the negatively charged phosphate backbone of the genetic material.^32,33^ PEGylation improves the extracellular stability when forming such polyplexes and reduces their cytotoxicity.^34^ At the same time, the degree of polymerization (DP) of the cationic block (PDMAEMA) influences the effectiveness of the polymer to condensate DNA through electrostatic interactions.^6^ The cationic block DP required for DNA complexation and gene delivery is highly studied but is dependent on the size of the genomic payload.^18,32^

Typically, these types of copolymers are synthesized using conventional reversible deactivation radical polymerizations, such as RAFT, that employ thermoinitiators that decompose at temperatures typically between 65 and 80 °C.^16,35,36^ RAFT polymerization is favored for its high control over the polymerization process and its ability to produce polymers with narrow molar mass distributions before they are mixed with DNA.^16,35^ Although this approach allows large batches of polymers to be produced, additional formulation steps require optimization and limit its suitability for rapid polymer screening. Before analyzing the polyplexes generated through PIESA in this work, a set of PEG_113_–PDMAEMA_*n*_ block copolymers, with DP_DMAEMA_ values of 60, 83, and 126, were synthesized via thermally initiated RAFT polymerization to serve as a controls.^18,32^ For ease of readability, all references to varying DP throughout this letter refer to the PDMAEMA block of the PEG_113_–PDMAEMA_*n*_ copolymers. Molecular weight data and GPC traces of the control polymers are provided in the Supporting Information (Table S1 and Figure S1).

To generate polyplexes in situ during polymerization and achieve a “one-pot” synthesis of the polyplexes, it was necessary to follow a different polymerization route and avoid the degradation of DNA from the high temperatures that are typically used in thermally-initiated RAFT polymerization. Instead, PET-RAFT polymerization was utilized to synthesize PEG_113_–PDMAEMA_*n*_ block copolymers (Figure 1a–d). The reaction mixture was prepared in water, incorporating ascorbic acid (AscA) as a reducing agent and acridine orange (AO) as a reducible dye to facilitate the generation of free radicals.^21^ This method is ideal for high-throughput sample preparation and screening, as it allows for faster, more streamlined operations in ultralow volumes (<200 μL) within multiwell plates without the need for extensive oxygen removal. AO was chosen due to its excitation wavelength, enabling the use of low-power visible light. This way, potential DNA damage associated with UV light exposure and the use of DMSO (commonly applied in other PET-RAFT systems) was avoided.^22,37^

**Figure 1:**
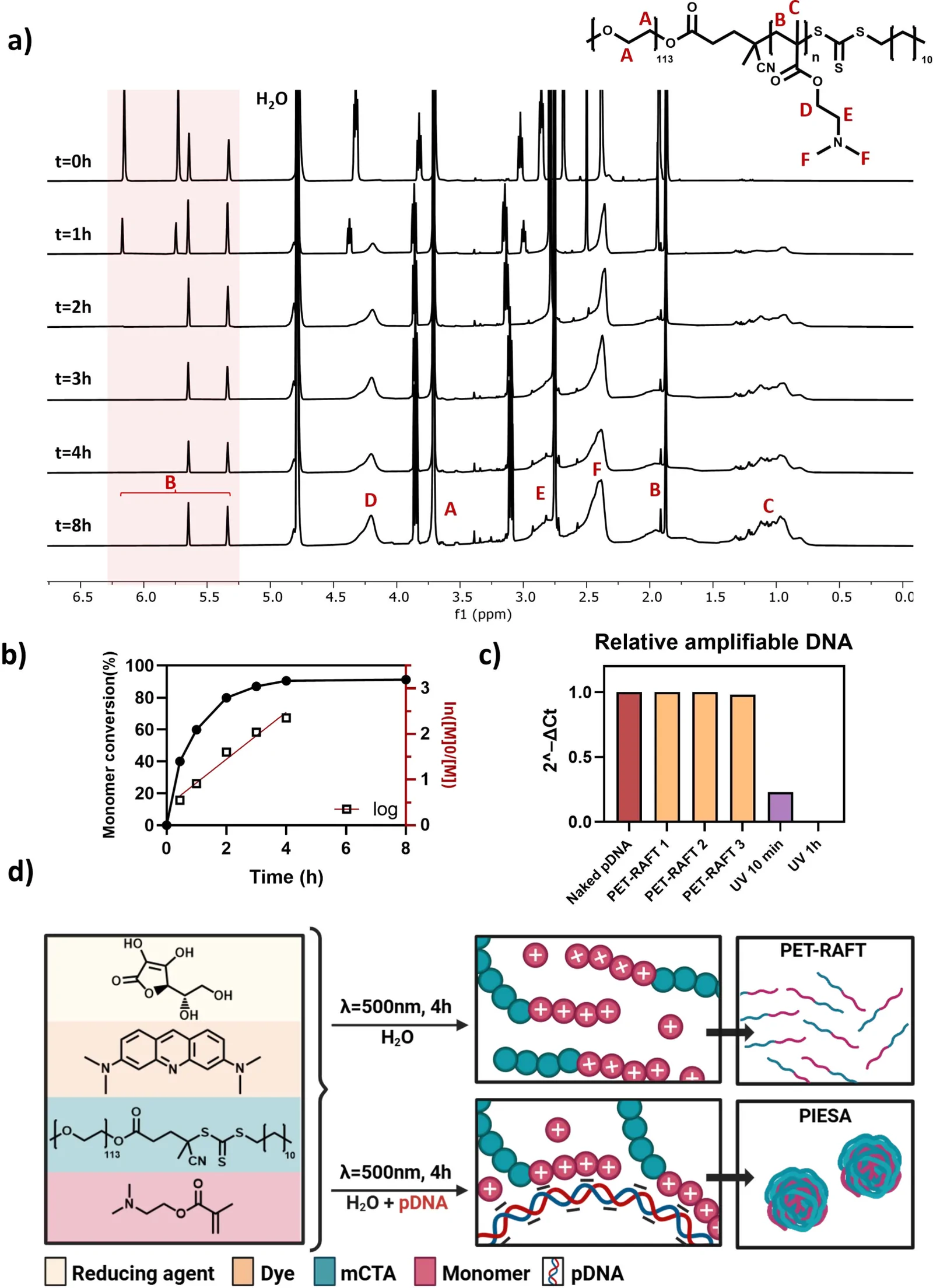
(a) ^1^H NMR spectra of PEG_113_–PDMAEMA_90_ synthesized via PET-RAFT polymerization measured in D_2_O (400 mHz) at different time points, showing the disappearance of the signal corresponding to the protonated vinyl peaks in the region of 5.3–6.2 ppm, reaching 90% monomer conversion after 4 h. (b) Monomer conversion calculated by ^1^H NMR at different time points and the corresponding semilog kinetic plot. (c) Quantitative PCR (qPCR) analysis of DNA following PET-RAFT and UV treatment, showing the relative DNA integrity expressed as 2^–Δ*C*_*t*_^, representing the fraction of amplifiable DNA remaining after treatment in relation to the untreated control. (d) PEG_113_–PDMAEMA_*n*_ block copolymer synthesis via PET-RAFT polymerization (top) and the synthesis and assembly of nanoparticles via PIESA (bottom).

The polymerization reaction was carried out in a 96-well plate (∼100 μL) and irradiated with an LED panel at λ ± 500 nm (Figure S2). Samples were collected at hourly intervals over 4 h, with a final sample taken at 8 h, to monitor monomer conversion by ^1^H NMR and evaluate the polymerization kinetics, thereby determining the optimal reaction end point (Figure 1a and Figure S2). The integral values of DMAEMA vinyl proton peaks revealed 90% monomer conversion after 4 h, which increased by only 1% after increasing the exposure time to 8 h (Figure 1b). The semilog kinetic plot showed a linear relationship (*R* ^2^ = 0.98), indicating first-order kinetics (Figure 1b). Based on this, the subsequent PET-RAFT reactions were stopped after 4 h of light irradiation. Simultaneously, molecular weight and molar mass dispersity were obtained using GPC to confirm successful block copolymer synthesis at the desired DP (Figure S3 and Table S2).

Having confirmed that PEG_113_–PDMAEMA_*n*_ block copolymers could be produced, we explored the addition of plasmid DNA (pDNA) to the reaction. The PIESA approach used was an adapted version of the PET-RAFT method described by Boyer et al.^21,38^ and the siRNA polyplexes developed by Shen et al.,^30^ enabling the assembly of biocompatible, readily available polymeric nanoparticles with pDNA (polyplexes) while preserving the integrity of the encapsulated cargo.

To confirm this, we assessed pDNA integrity using quantitative PCR (qPCR), based on amplification efficiency (Figure 1c). PET-RAFT polymerization of a noncharged monomer was carried out in the presence of mCherry pDNA to replicate the conditions that will be used for PIESA. An unreacted PET-RAFT solution containing mCherry pDNA served as an untreated control, while matching solutions exposed to UV light (for 10 min and 1 h) were included as damage-positive controls. Following sample purification, qPCR was performed, generating an amplicon from the mCherry gene. Comparison of cycle threshold (*C*_*t*_) values showed no significant differences between the PET-RAFT-treated samples and the untreated control (Figure S4). In contrast, the positive control UV-treated samples showed Δ*C*_*t*_ values of 2 and 11, corresponding to 25% and 0.04% of the original amplifiable DNA remaining, respectively (Figure 1c and Table S3). These results demonstrate that pDNA is not harmed by the PET-RAFT based PIESA approach.

To prevent DNA from post-PIESA degradation and facilitate the formation of polyplexes suitable for transfection, the copolymer to DNA concentration was evaluated in terms of N/P ratio, which represents the molar ratio of amine groups (N) in the cationic copolymer to phosphate groups (P) in the DNA backbone.^39^ N/P ratios typically between 5 and 40 have previously been shown to create polyplexes with no free DNA.^40^ Therefore, using a pDNA containing mCherry fluorescent reporter gene (to track transfection in future steps), we explored N/P ratios of 10, 20, and 30 with PEG_113_–PDMAEMA_*n*_ (where *n* = 60, 90, or 120) using a PET-RAFT based PIESA approach (Figure 1d).

For effective transfection, polyplex electrostatic binding strength,^41^ overall charge,^42,43^ and particle size^44^ are crucial factors to consider. To confirm polyplex formation after PIESA, the presence (or absence) of free DNA was investigated. Free DNA is unable to cross cellular membranes due to its size and negative charge,^45^ whereas complexation enhances gene delivery and expression.^46^ DNA complexation was evaluated by agarose gel electrophoresis, where free nucleic acids migrate similarly to naked DNA, whereas complete complexation is indicated by the retardation of DNA migration.^47^ The control PEG_104_–PDMAEMA_*n*_ block copolymers prepared via conventional thermal RAFT polymerization were mixed with pDNA at N/P ratios of 10, 20, and 30. These were referred to as conventional polyplexes and serve as a reference to compare with polyplexes assembled via PIESA at the same N/P ratios.

Conventional and PIESA-derived polyplexes were analyzed by agarose gel electrophoresis, with each well containing 1 μg of DNA. Variations in polymer molar mass and charge density can influence how the polymers interact with the DNA, resulting in polyplexes with differing degrees of complexation or stability.^48^ Regardless of the assembly method, all formulations decreased the electrophoretic mobility of the pDNA band, indicating polymer/pDNA complexation (Figure 2a). In most cases, the absence of smearing indicated successful complexation. However, PIESA formulations at low DP (60) and low N/P (10) showed slight smearing, suggesting partial complexation and the need for higher polymer content.^47^ Similarly, at DP120 and N/P10, multiple bands were observed, indicating populations with weaker DNA binding (Figure 2a). In both cases, complexation improved at higher N/P ratios (20 and 30), as evidenced by the disappearance of smears.

**Figure 2:**
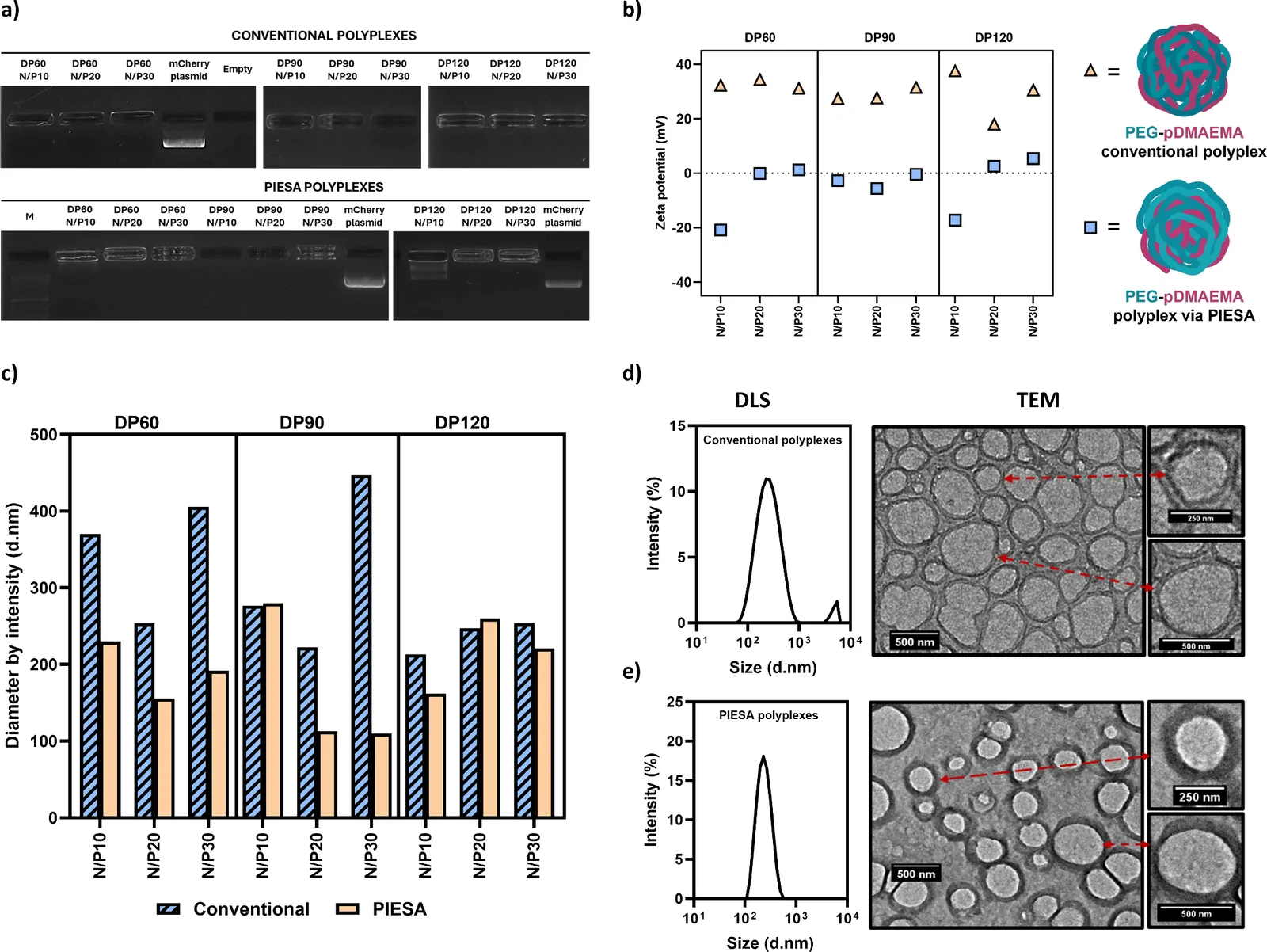
Physicochemical characterization of polyplexes at varying DP_DMAEMA_ and N/P ratios. (a) Agarose electrophoresis: retardation of various polyplex formulations, each containing 1 μg of plasmid DNA (pDNA). Lane “M” displays the 1 kb Plus DNA Ladder for reference, while the “mCherry plasmid” lane shows 1 μg of naked pDNA. (b) Zeta potential of polyplexes in 0.1 mM KCl (pH ∼ 6.5). (c) Hydrodynamic diameter of polyplexes with different formulations measured by DLS. (d, e) Hydrodynamic diameter distribution by intensity measured with DLS and TEM images comparing conventionally assembled and PIESA-derived polyplexes at DP90 N/P 10.

Once DNA binding was confirmed for all formulations, the properties of the resulting polyplexes were investigated. Analysis of the surface charge characteristics of nanoparticles in solution revealed distinct differences between polyplexes synthesized via conventional methods and those obtained through PIESA (Figure 2b and Table S4). Thermo-RAFT-derived polyplexes displayed a Zeta potential greater than +30 mV at pH ∼ 6.5, except for the DP120 polyplexes at N/P 20 with +18 mV. This is indicative of protonated amine groups on the nanoparticle surface and possibly a randomized assembly.

Polyplexes synthesized using PIESA generally exhibited a slightly negative to neutral Zeta potential at pH ∼ 6.5. Specifically, at N/P 10, formulations with the lowest (DP60) and highest (DP120) polymer molecular weight reached a Zeta potential of −20 mV (Figure 2b). The remaining formulations displayed a near-neutral Zeta potential, with values ranging from −5 to +5 mV, which is characteristic of efficient surface shielding by the PEG block. Such a surface charge is advantageous for systemic applications, as it may contribute to enhanced colloidal stability,^49^ prolonged circulation time in vivo,^50^ and reduced cytotoxicity.^51,52^ Overall, the Zeta potential of PIESA-derived polyplexes was slightly more positively charged with high N/P ratios, which was not a trend for the conventional nanoparticle assembly. The Zeta potential values observed for DP60 and DP120 at N/P 10 correlate with migration patterns seen in the electrophoretic retardation assay (strongly negative, migrating toward the positive pole), confirming our previous assumptions that at this N/P ratio, a DP of 90 is necessary to fully complex and neutralize the net negative potential of the plasmids. Therefore, at DP90, the optimal number and molar mass of block copolymer chains in PIESA polyplexes are achieved, providing an optimal equilibrium for effective neutralization.

Further characterization of polyplexes was assessed by dynamic light scattering (DLS), revealing that particle hydrodynamic diameters were influenced by the DP, N/P, and assembly method used (Figure 2c). Certain formulations displayed a tendency to aggregate across both assembly methods, suggesting challenges in maintaining electrostatic stabilization at these polymer DPs (Figure S5). The presence of polyplex aggregates is an important consideration in gene delivery applications, as large, stable aggregates can hinder cellular uptake and intracellular trafficking. However, Roy et al.^53^ previously reported that even aggregated polyplexes can mediate efficient transfection. These aggregates could potentially represent “soft clusters” characterized by low electrostatic binding strength, which might allow them to dissociate upon internalization and facilitate nucleic acid release.

Polyplexes at DP60 and DP90 showed substantial differences in hydrodynamic diameters between assembly methods (Figure 2c). PIESA formulations decreased in size with increasing NP ratio, indicating enhanced DNA complexation. Together with the absence of aggregates at DP90, this suggests an optimal balance between polymer molar mass and electrostatic interactions, resulting in uniform, compact complexes with improved colloidal stability. The opposite trend was observed for the conventionally assembled polyplexes at this DP, where larger polyplexes were obtained with a higher N/P ratio. Overall, conventional polyplexes assembled with lower molar mass polymers yielded larger particles (∼400 nm), whereas those assembled at higher DPs resulted in smaller particles (∼240 nm), underscoring the influence of polymer chain DP on particle size. At DP120, both methods yielded similar particle diameters (200–250 nm) (Figure 2c). As a final step, the stability of PIESA and conventionally assembled polyplexes (DP90 and N/P10) was monitored by DLS over 7 days, with both formulations remaining stable throughout the measurement period (Figure S6).

To better understand the polyplex morphology and size, TEM imaging was performed on polyplexes prepared at the optimal DP (90) and the lowest N/P ratio. Both types of polyplexes exhibited populations with sizes close to those reported by DLS and appeared to have similar quasi-spherical morphologies after being dried and stained (Figure 2d,e). Conventional nanoparticles were characterized by smooth yet slightly irregular contours, indicative of minor asymmetry in shape (Figure 2d). PIESA polyplexes, while similar, displayed smooth and predominantly symmetrical contours with minimal deviations from roundness (Figure 2e). For both assembly approaches, some nanoparticles appeared larger in TEM than in DLS, as DLS measures particles in their hydrated state, whereas TEM involves drying, which can cause deformation or flattening.^54^

Evidence of polyplex merging during sample preparation was also observed (Figure 2d and Figure S7). Nonetheless, many particles exhibited dimensions consistent with the DLS data (Figure 2c–e). These results confirm the formation of stable polyplexes by both methods, with particle size governed by polymer DP and assembly strategy. While the N/P ratio modulates electrostatic interactions,^55^ the polymer DP more significantly governs DNA condensation.

Previous studies have shown that siRNA can be successfully packaged using PIESA,^30^ but siRNA is a relatively small (∼13 kDa), primarily single-stranded molecule. In contrast, a 6 kb plasmid (∼3,900 kDa) is a significantly larger, rigid, circular double-stranded structure. The ability of PIESA to effectively complex such large plasmids while preserving DNA integrity significantly expands the scope of PIESA for nonviral gene delivery applications. To assess cellular uptake, PIESA polyplexes (DP90 and N/P10) were prepared using DiTO-3-labeled pDNA and applied to Vero cells. Following synthesis, polyplexes were diluted in serum-free DMEM, incubated for 30 min, and added to cells. After 4 h at 37 °C, cells were washed, fixed, stained, and analyzed by confocal microscopy. Fluorescence imaging showed the predominant nuclear accumulation of labeled pDNA (Figure 3). To confirm the intracellular localization and distinguish between surface-bound and internalized polyplexes, Z-stack imaging was performed, demonstrating that the fluorescence signal was located within the cytoplasm and at the level of the nucleus, rather than on the cell surface (Figure 3).

**Figure 3:**
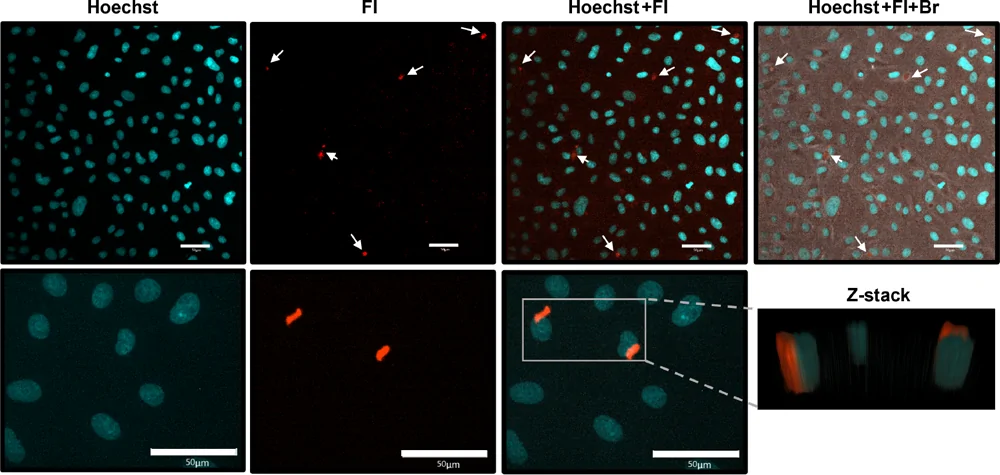
Confocal fluorescence microscopy images of Vero cells following transfection with PIESA-derived polyplexes at DP90 and N/P 10. Cells were imaged 4 h post-treatment with polyplexes carrying fluorescently-labeled pDNA. 10× (first row) and 20× (second row) magnification. Scale bar = 50 μm.

Following confirmation of intracellular delivery, the transfection efficiency of PIESA polyplexes was evaluated with pDNA molecules carrying an mCherry reporter gene (not labeled) driven by the constitutively active CMV promoter. Polyplexes were added at pDNA doses of 0.7–2 μg and incubated at 37 °C for 48 h before flow cytometry analysis. The percentage of mCherry-positive cells and mean fluorescence intensity (MFI) were used to assess transfection, while DAPI staining quantified cell viability. PIESA-derived polyplexes maintained high viability (>85%) across all conditions (Figure 4a), indicating improved biocompatibility compared to conventional formulations. In contrast, conventional polyplexes showed high viability only at low DNA doses and DP60, but this decreased sharply with increasing N/P ratio and DNA concentration, reaching ∼20% at N/P 20 and <10% at N/P 30 (Figure 4a,b). This increased cytotoxicity also translated to reduced transfection efficiency and is consistent with stronger electrostatic interactions between highly cationic polyplexes and cell membranes, leading to membrane disruption.^56,57^

**Figure 4:**
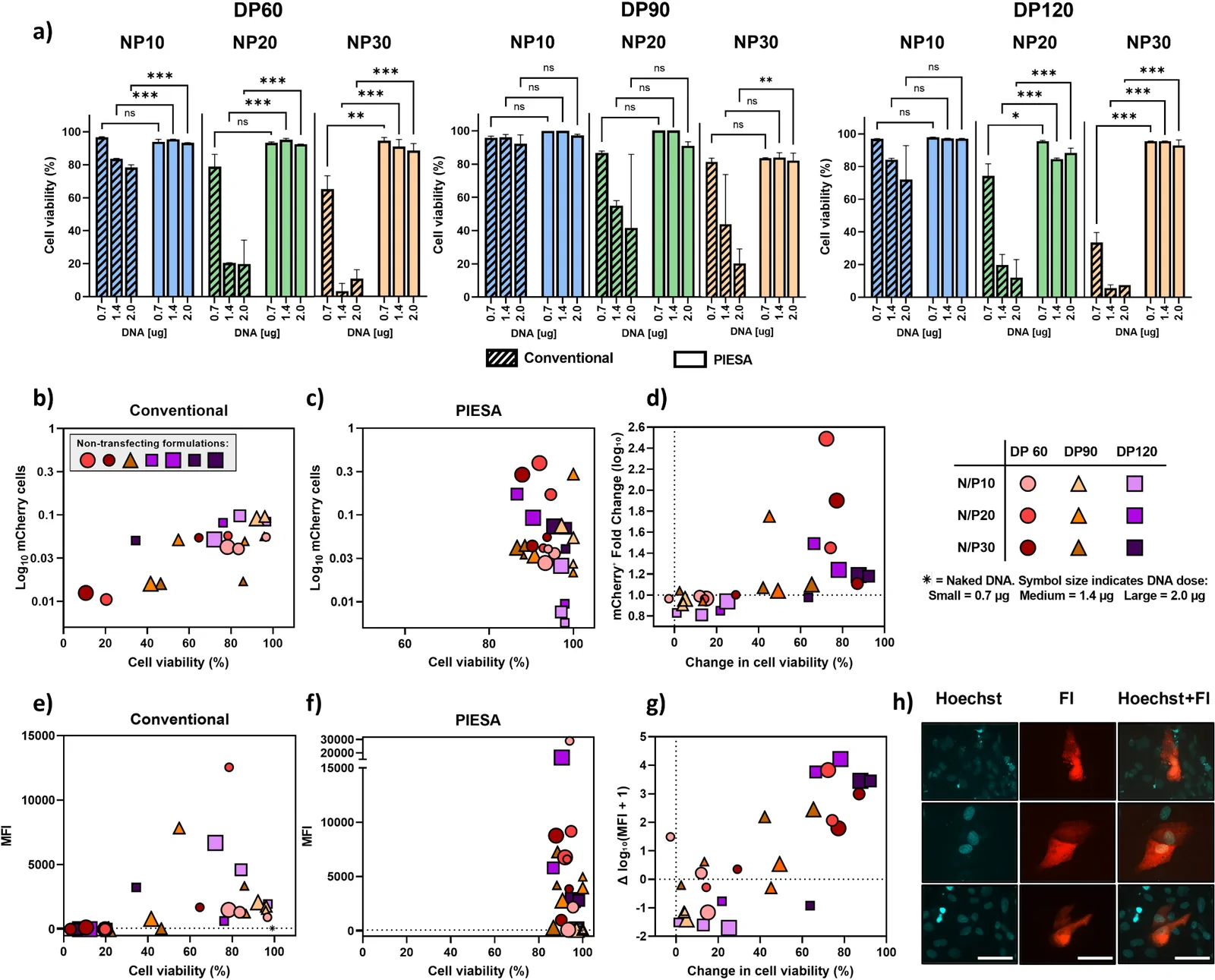
(a) Comparison of cell viability following transfection with conventional and PIESA polyplexes across all formulations and DNA doses. Statistical analyses were performed using two-way ANOVA (*p < 0.05, **p < 0.01, ***p < 0.001, ns = nonsignificant). (b–g) Flow cytometry analysis of the transfection efficiency in relation to the cytotoxicity of each polyplex formulation. Each symbol represents an individual formulation, where the shape indicates the polymer DP (circles, triangles, or squares), the shade reflects the N/P ratio (darker color indicates a higher N/P ratio), and the symbol size corresponds to the DNA dose carried (0.7, 1.4, or 2 μg per well). The relative transfection efficiency or mean fluorescent intensity (MFI) of cells expressing mCherry is presented on the *Y*-axis. (d) Scatter plot showing the fold change for each sample (conventional vs PIESA) calculated as 10^(log_10_ mCherry cells conventional–PIESA)^. The dashed line at fold = 1 indicates no change between conditions; points above the line mean PIESA is greater, while points below mean it is lower. (g) MFI log difference values represented as Δlog_10_ = log_10_(MFI_PIESA_ + 1) – log_10_(MFI_conventional_ + 1) to avoid zeros. The dashed line at fold = 1 shows no change. Higher values show strong increases in the fluorescence signal in PIESA. (h) Confocal fluorescence microscopy images of Vero cells (20×) 48 h following transfection with PIESA-derived polyplexes at DP90 and N/P 20 containing mCherry plasmid. Scale bar = 50 μm.

To benchmark the performance, the conventionally assembled polyplexes were first evaluated. As reported for PEG–PDMAEMA systems, optimal transfection occurs at approximately N/P 10, with higher ratios increasing toxicity.^18,34^ Consistent with this, conventional polyplexes transfected at low N/P ratios but showed limited improvement at higher ratios. Increasing the DNA dose or N/P ratio led to reduced viability and lower mCherry expression (Figure 4b), suggesting less predictable performance due to uncontrolled assembly. Instead, using PIESA, it was possible to assess whether higher N/P ratios and higher polymer DPs could enhance transfection while minimizing cytotoxic effects. Notably, it was demonstrated that N/P ratios as high as 20–30 were not only well-tolerated by the cells but also achieved significant benefits in transfection efficiency compared to conventional polyplexes (Figure[Fig fig4]c). This shift suggested that alternative assembly methods could unlock the potential for high polymer ratios. Formulations with moderate to high N/P ratios and DNA doses tended to cluster in the upper right side of the graph in Figure 4c, indicating a favorable balance between efficient gene delivery and minimal cytotoxicity. For example, DP60 formulations improved significantly with PIESA, as higher N/P ratios had a 1.9–2.6-fold increase in transfection levels and cell viability increased more than 70% (Figure 4d).

In addition, formulations that did not transfect when assembled conventionally showed markedly high MFI values when prepared using PIESA, in some cases exceeding the MFI levels of the best conventional formulations (Figure 4f,g). For example, the log_10_-transformed MFI for DP90 formulations at high DNA doses increased by approximately 2 units, reflecting a 100-fold improvement (Figure 4g). Similarly, DP120 formulations that were previously incapable of transfection displayed a 4 unit increase in log_10_ MFI, corresponding to a 100,000-fold enhancement. These results highlight the potential of PIESA to access more robust polymer–DNA interactions that enhance cellular uptake and gene expression.

Increasing the plasmid dose did not always result in proportional increases in mean fluorescence intensity (MFI) (Figure S9), indicating saturation of transgene expression. For PIESA formulations at N/P 20, a plateau was reached, consistent with previous reports showing saturation at high pDNA concentrations.^58^ This behavior suggests that the delivered pDNA remained intact, as degraded DNA would reduce expression rather than plateau. Saturation in conventional polyplexes was observed only at N/P 10, likely due to the cytotoxicity of higher N/P formulations. Notably, aggregated PIESA polyplexes (e.g., DP120) retained transfection capability, indicating that aggregation did not compromise DNA integrity or delivery efficiency.

It is important to note that not all formulations benefited equally from PIESA. Polyplexes at low N/P ratios showed minimal changes in transfection efficiency or cytocompatibility, likely due to their inherent stability and low toxicity. Consequently, improvements in viability were modest (1–20%). These results indicate that while PIESA enhances formulations limited by stability or efficacy, its impact is less pronounced for already optimized systems.

PEG_113_–PDMAEMA_*n*_ polyplexes assembled via PIESA achieved transfection efficiencies comparable to those of conventional controls with minimal cytotoxicity (Figure 4a–h), consistent with previous reports,^59^ as PEG–PDMAEMA is known for low transfection efficiency due to the shielding effect of PEG.^60^ Nevertheless, such levels are suitable for ex vivo applications where cell enrichment techniques are routinely employed.^61^ The cytocompatibility of this platform provides opportunities for further optimization, including stimuli-responsive PEGylation (e.g., acid-labile or cleavable bonds) or alternative hydrophilic stabilizers such as polyzwitterions or polyoxazolines.^62–65^

Overall, PIESA polyplexes exhibited a broader window of effectiveness due to their assembly approach. These results underscore how PIESA can control the synthesis and physicochemical properties of polyplexes and improve the performance of cationic gene delivery systems. It demonstrates strong potential as a versatile platform for designing polyplexes that can overcome biological barriers and advance future gene delivery strategies. PIESA has the ability to safely use higher N/P ratios and polymers with higher molar mass without compromising cell viability and enabling higher DNA payloads, which represents a significant advance in the generation of gene delivery systems. These findings strongly suggest that conventional assembly methods may not fully exploit the potential of polymers for biological applications. Transitioning to advanced synthesis and assembly strategies such as PIESA could unlock next-generation gene delivery systems with superior transfection, broader formulation windows, and improved cytocompatibility.

## Supplementary Material



## Notes

Additional data to support the characterization of block copolymers (^1^H NMR spectroscopy as well as GPC traces and analysis), qPCR analysis (primer sequences and raw data), polyplex characterization (DLS traces of polyplexes and TEM images), and flow cytometry analysis of transfected cells (PDF)
