# Supplementary material for: Polymerization-Induced Electrostatic Self-Assembly Enables Noncytotoxic Polyplex Formation for Gene Delivery
Source: ACS Mater Lett. 2026 Apr 15;8(8):2139–47. doi: 10.1021/acsmaterialslett.6c00077 (PMC13580189; doi:10.1021/acsmaterialslett.6c00077)
Supplement: Supplementary file 1 [file tz6c00077_si_001.pdf]

Polymerization-Induced Electrostatic Self-Assembly Enables Non-Cytotoxic Polyplex Formation for Gene Delivery

Maria A. Castillo,[†,‡] Jungyeon Kim,[†,‡] Francesca Patel-Burrows,[†,‡] Xueyuan Li,[†,‡] Antony Adamson,[¶] Lee A. Fielding,[*,†,‡] and Samuel T. Jones[*,§,†]

†*Department of Materials, University of Manchester, Manchester, M13 9PL*

‡*Henry Royce Institute, University of Manchester, Manchester, M13 9PL*

¶*Genome Editing Unit, University of Manchester, Manchester, M13 9PL*

§*School of Chemistry, University of Birmingham, Edgbaston, B15 2TT*

E-mail: lee.fielding@manchester.ac.uk; s.t.jones.1@bham.ac.uk

Supporting Information Available

Experimental section

Materials

Poly(ethylene glycol) methyl ether (4-cyano-4-pentanoate dodecyl trithiocarbonate) 5400 Mw, 2- (Dimethylamino)ethyl methacrylate (DMAEMA), 2-hydroxyethyl acrylate, 4-((((2-carboxyethyl)thio)carbonothioyl)thio)-4-cyanopentanoic acid, 3,6-Bis (dimethylamino) acridine (acridine orange) and L-Ascorbic acid (AscA) were obtained from Sigma Aldrich. DMAEMA liquid monomers were disinhibited by passing through a column of basic alumina prior usage in any reaction. Dulbecco's Modified Eagle Medium (high glucose DMEM),

Opti-MEM™ I Reduced Serum Medium, 50X Tris-acetate-EDTA (TAE) buffer, SYBR® Safe DNA gel stain, 1Kb plus DNA ladder, Fetal Bovine Serum (FBS) were purchased from Thermo Fisher Scientific - Invitrogen. The mCherry expression plasmid was constructed by cloning the mCherry gene from the mCherry2-C1 plasmid (Addgene plasmid 54563) into the pcDNA™3.1 backbone (Addgene, USA). DiTO™-3 labeling dye was obtained from AAT Bioquest.

Synthesis of PEG_{113}-$PDMAEMA_n$ block copolymers via thermally initiated RAFT polymerisation

Polymerisation mixture was prepared at a molar ratio of [DMAEMA] : [PEG mCTA] : [ACVA] = [n] :[1] : [0.1] and dissolved in DMF to a final concentration of 20 % w/w. The solution was degassed under nitrogen for 30 minutes and polymerised overnight at 70 °C. The reaction was quenched on ice, diluted with THF and precipitated three times into cold hexane via centrifugation at 4200 rpm. The resulting block copolymers were collected and lyophilised.

PET-RAFT polymerisation

The synthesis of PEG_{113}-$PDMAEMA_n$ block copolymers via PET-RAFT polymerisation was carried out in a 96-well plate using PEG_{5400} as a mCTA, acridine orange base was used as a photocatalyst and ascorbic acid as a reducing agent. PET-RAFT reactions were prepared in 96-well plates at a ratio of [monomer] : [mCTA] : [acridine orange] : [AscA] = [n] : [1] : [0.05] : [1]. Mixtures were dissolved in deionised water (final volume up to 200 μL) and irradiated by a LED lamp ($\lambda \pm 500$ nm) for 4 hours at room temperature.

PET-RAFT polymerisation of 2-hydroxyethyl acrylate was carried out in deionised water (final volume: 100 μL) using 4-((((2-carboxyethyl)thio)carbonothioyl)thio)-4-cyanopentanoic acid as the chain-transfer agent (CTA), to assess pDNA integrity via qPCR. The reaction mixture was prepared with the following molar ratios: [monomer] : [CTA] : [acridine orange]

: [AscA] = [120] : [1] : [0.05] : [1].

qPCR-Based assessment of pDNA integrity

Plasmid DNA integrity was evaluated by quantitative PCR (qPCR) based on amplification efficiency. PET-RAFT polymerisation of 2-hydroxyethyl acrylate was performed in the presence of mCherry pDNA to replicate PIESA conditions. An unreacted PET-RAFT mixture served as a control and a UV-exposed sample as a damage-positive control. Samples were purified using the QIAquick PCR Purification Kit (Qiagen). qPCR reactions (20 μL) contained PowerUp™ SYBR™ Green Master Mix (Thermo Fisher), 50 ng plasmid DNA, and 400 nM primers targeting a 152 bp region of mCherry (forward: 5'-GAGATCAAGCAGAGGCTGAA-3'; reverse: 5'-ATGGTGTAGTCCTCGTTGTG-3'). The treated condition was prepared in triplicate (independent reaction tubes), each of which was run in technical triplicate by qPCR. Control samples (untreated and UV-treated) were prepared once and run in qPCR triplicates. Data are presented as mean of qPCR replicates. Amplification was performed on a StepOnePlus™ system (Applied Biosystems) (95 °C, 2 minutes; 40 cycles of 95 °C for 15 seconds and 60 °C for 1 minute). C_t values were used to calculate ΔC_t (ΔC_ttreated – ΔC_tcontrol) and relative DNA integrity was determined using the $2^{-\Delta C_t}$ method.

^{1}H NMR analysis

The products were analysed by proton nuclear magnetic resonance spectroscopy (^{1}H NMR) on a Bruker Avance III 400MHz spectrometer with 128 scans averaged per spectrum and raw data was processed using MestreNova (v14.2.1-27684). DMAEMA monomer conversion was calculated by comparing the integral of the polymer peak at 1.9 ppm to the sum of this polymer peak and the vinyl proton peaks of unreacted DMAEMA monomer (6.2 ppm, 1H; 5.7 ppm, 1H; 5.6 ppm, 1 H; 5.3 ppm, 1H). The PDMAEMA DP was calculated by comparing the integral of the PEG backbone peak (internal reference at 3.6 ppm) to the integral of the methylene peak of the PDMAEMA side chain at 1.9 ppm and the amine proton environment

at 2.3 ppm.

Gel Permeation Chromatography

Samples run in DMF were analysed using an Agilent 1260 Infinity II GPC system equipped with a refractive index detector and two PLgel 5 μm Mixed-D columns. The mobile phase consisted of HPLC-grade dimethylformamide (DMF) with 10 mM lithium bromide, operated at 60 °C and a flow rate of 1 mL min^{-1}. The DMF GPC system was operated without a guard column as part of the standard instrument configuration. Molar mass distributions were determined using poly(methyl methacrylate) (PMMA) standards for calibration (540 to 44,760 g mol^{-1}). Additional samples were run in dimethylacetamide (DMAc) on a separate Agilent 1260 Infinity SEC system equipped with differential refractive index (DRI), viscometry (VS), dual-angle light scattering (LS), and UV detectors. This setup included two PLgel Mixed-C columns (300 × 7.5 mm) and a PLgel 5 μm guard column, with 0.45 wt% LiCl in DMAc as the eluent, operated at 0.8 mLmin^{-1} and 60 °C. PMMA standards 550 to 955,000 g mol^{-1}) were also used for calibration. All samples were filtered through 0.2 μm PTFE filters before injection.

Polyplex generation via PIESA and conventional assembly

To perform PIESA, the same reagents and conditions described in the PET-RAFT section were used, maintaining the [monomer] : [mCTA] : [acridine orange] : [AscA] ratios. The key difference was the addition of pDNA in varying N/P ratios at the same time as the rest of the reagents. The N/P ratios were calculated using the formula *N/P = (mol N) / (mol P)* where 'mol N' is the number of moles of protonatable nitrogen from the reagent in the transfection mixture, and 'mol P' is the number of moles of anionic phosphate from the nucleic acid in the transfection mixture. The reaction was prepared with 1% w/w of polymer in of molecular-grade water. The mixture was irradiated at $\lambda \pm 500$ nm for 4 hours and used for transfection on the same day. For conventional polyplex assembly, the block copolymers

were mixed with pDNA in molecular-grade water at different N/P ratios and incubated at room temperature for one hour prior transfection.

Gel Electrophoresis

Agarose gels for electrophoresis were prepared with 1% agarose, 1X TAE buffer and SYBR® Safe DNA gel stain (1:10000). Agarose gels were loaded with 1Kb plus DNA ladder in loading buffer and samples were diluted 1:6 volume in loading dye. The DNA and polyplexes were resolved at 75 V, 40 minutes and analysed under UV light.

Dynamic Light Scattering

Polyplex samples were analysed by dynamic light scattering (DLS) using a Malvern Zetasizer Ultra instrument (Malvern instruments, UK) with a cumulative fit correlation at 25 °C. The hydrodynamic diameter (D_h), polydispersity index (PDI) and Z-potential were obtained using the ZS Xplorer software V3.1.064 (Malvern Panalytical Ltd., UK). The polyplexes were resuspended in deionised water and diluted in 0.1 mM KCl (pH $\sim$ 6.5) to 0.05% w/w and loaded in Malvern Panalytical Folded Capillary Zeta Cells (DTS1070). Acquired data were averaged over three consecutive runs and the hydrodinamic diameter (D_h) values were reported. The data was plotted using GraphPad Prism v 9.3.1 (GraphPad Software, USA).

Transmission Electron Microscopy

Nanoparticle-DNA dispersions were diluted to 0.0125-0.025% w/w at 20 °C prior to staining. 400 mesh carbon-coated copper grids were treated with plasma ash for 60 seconds, then 10 uL of sample was placed onto the grids and left to dry overnight. The samples were stained by placing 10 μL of 1% w/w aqueous uranyl acetate onto the grids which was blotted away with filter paper after 20 seconds. The grids were dried in air for at least 30 minutes before imaging. Imaging was performed using a F20 Tecnai TEM instrument operating at 200 kV and equipped with a Gatan Orius SC200D CCD camera in brightfield.

Cell Culture

African green monkey kidney (Vero) cells (ATCC (CCL-81)) were plated 24 hours before transfection on a flat bottom 24-well plate in 500 μL DMEM, supplemented with 10% FBS and 1% penicillin-streptomicin so that cells were 70-80% confluent at the time of transfection.

Transfection

To evaluate the gene delivery efficiency of polyplexes, previously plated Vero cells were washed with PBS and the culture media was replaced with clear DMEM (without antibiotics or FBS). The generated polyplexes (PIESA or conventional) were resuspended in clear DMEM, incubated 30 minutes at room temperature and added to the cells dropwise. A negative control was carried out adding naked mCherry pDNA to the cells. The plate was gently rocked back and forth, then incubated for 48 hours at 37 °C and 5% CO_2.

To monitor the intracellular localisation of PIESA polyplexes, plasmid DNA (pDNA) was fluorescently labelled using DiTO™-3 (5 mM in DMSO) at a base pair-to-dye ratio of 300:1, following the manufacturer's instructions. The pDNA was incubated with the dye at room temperature for 30 minutes in the dark, then used directly for PIESA polyplex assembly. PIESA polyplexes containing labelled pDNA (1.4 μg per well) were incubated with cells for 4 hours, followed by PBS washes and nuclear staining with DAPI prior to confocal microscopy analysis.

Confocal microscopy

A CellVoyager CQ1 Benchtop High-Content Analysis System was used for Cell imaging using the following channels: excitation = 651 nm, emission = 617/73 (red); lamp for bright field: 4X and 10X dry objective lenses.

Flow cytometry

Forty-eight hours post-transfection with either PIESA or conventional polyplexes, Vero cells cultured in flat-bottom 96-well plates were harvested for flow cytometric analysis. Cells were enzymatically detached using 0.25% trypsin and centrifuged at 400 × g for 5 minutes. The resulting pellets were washed twice with FACS buffer (1× PBS, 1% BSA, 25 mM EDTA) to remove residual enzymes and debris. To assess cell viability, DAPI (0.7 μg/mL) was added to the cell suspension and incubated for 10 minutes at room temperature in the dark. After one final wash, cells were resuspended in PBS for acquisition. Flow cytometric analysis was performed in triplicates using a cytometer equipped with U450/50-A and Y610/20-A filter channels. These settings enabled the identification of three distinct cell populations: DAPI-positive dead cells, mCherry-negative viable cells, and mCherry-positive transfected cells. Both population frequencies and mCherry fluorescence intensity were quantified to evaluate transfection efficiency and expression levels.

Table S1. GPC Characterisation results of PEG_{113}-$PDMAEMA_n$ polymers synthesised via thermally initiated RAFT polymerisation.

Polymer	M_{ntheo}	$M_{nGPC}{}^a$	Đ
PEG_{113}-$PDMAEMA_{60}$	14,800	23,500	1.08
PEG_{113}-$PDMAEMA_{83}$	18,500	24,300	1.11
PEG_{113}-$PDMAEMA_{126}$	25,200	29,200	1.14

[a] Mn_{theo} was calculated by adding the known $M_{wPEG113}$ to the theoretical $M_{wDMAEMA}$ multiplied by the DP calculated by ^{1}H NMR.

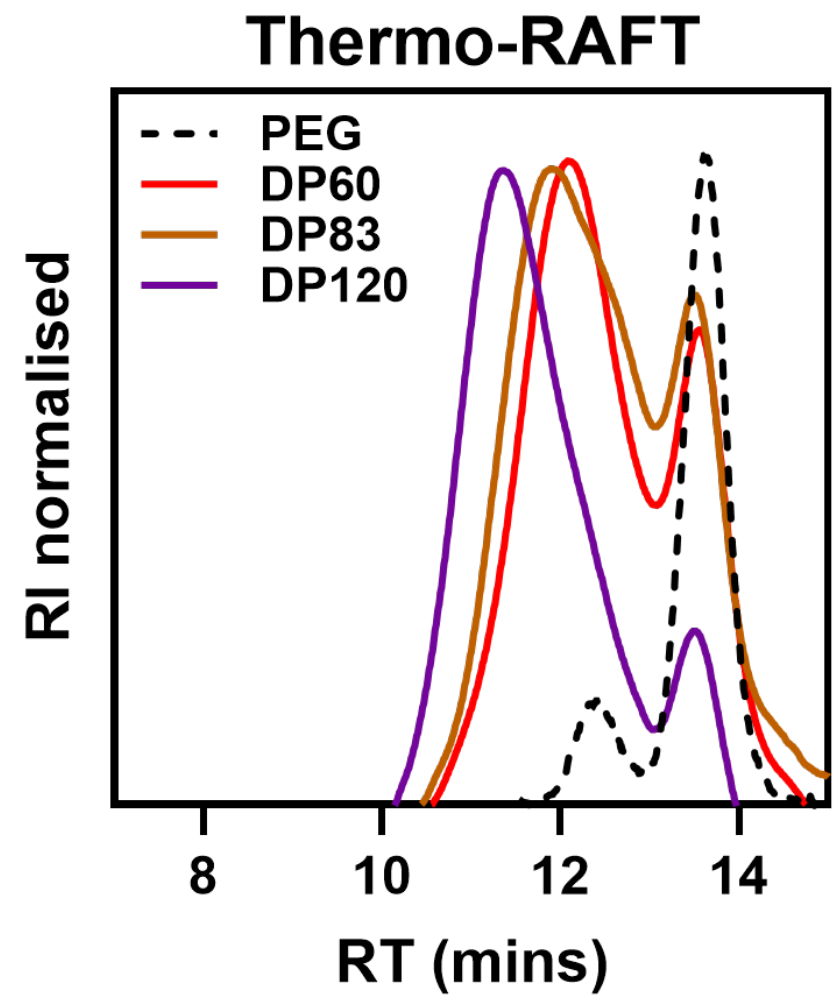

Figure 1: Figure S1: GPC traces of PEG_{113} mCTA and PEG_{113}-$PDMAEMA_{60,83,126}$ block copolymers in DMF. The shift of the curves towards the lower retention time are indicative of larger molecules and corresponded to increasing DP. The smaller curve observed at 13.5 minutes was consistent with the impurities present in the original PEG_{113} mCTA and found by other authors.[1]

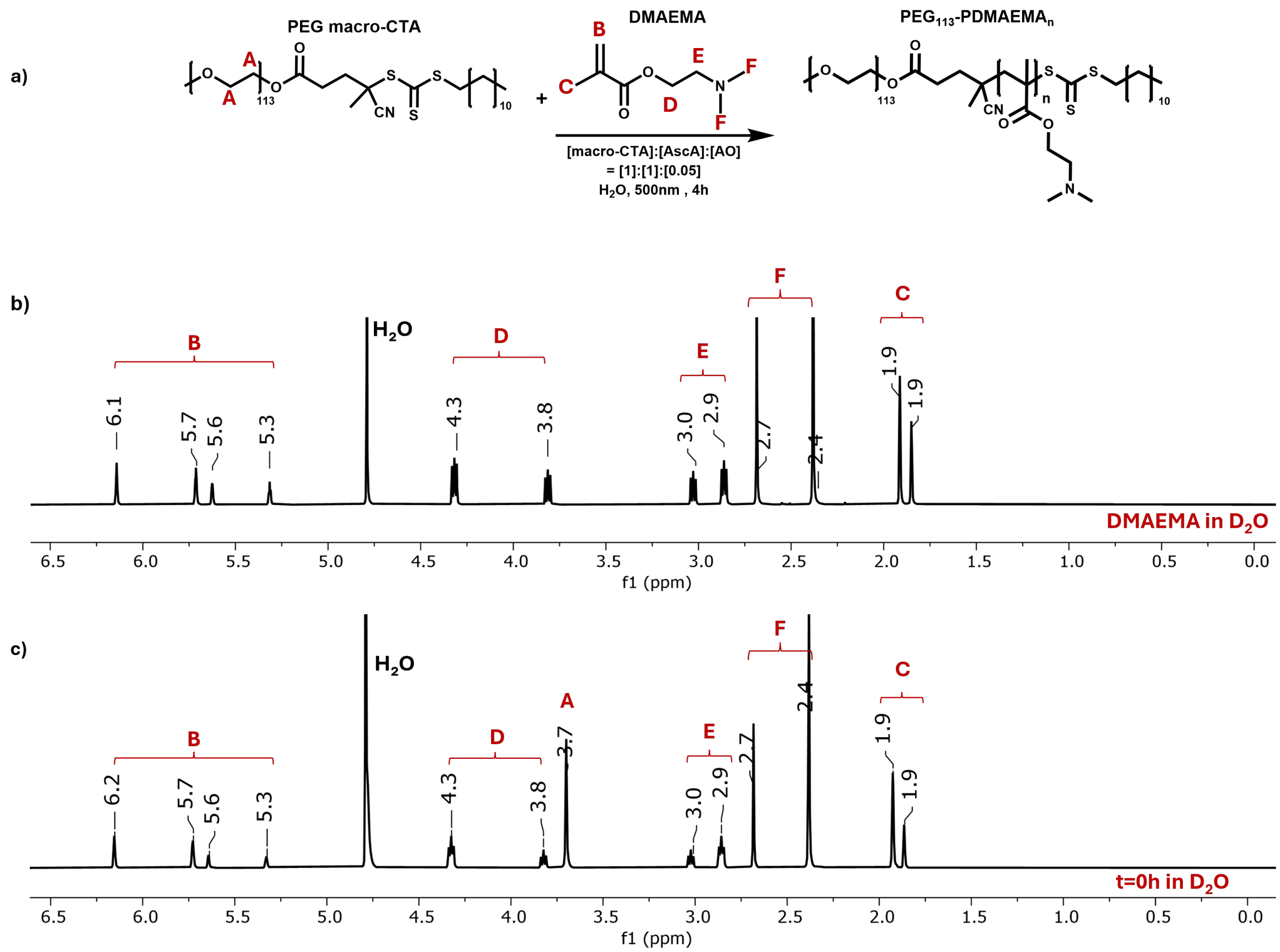

Figure S2: a) Reaction scheme of PEG_{113}-$PDMAEMA_n$ synthesis via PET-RAFT polymerisation. b) ^1H NMR spectrum of DMAEMA in D_2O showing a duplication of peaks. For example, the vinyl proton signals observed at 6.1, 5.7 ppm, 5.6 and 5.3 ppm corresponds to protonated and non-protonated species present at near neutral pH. c) ^1H NMR spectrum of PEG_{113}-$PDMAEMA_n$ synthesis via PET-RAFT before polymerisation (t=0).

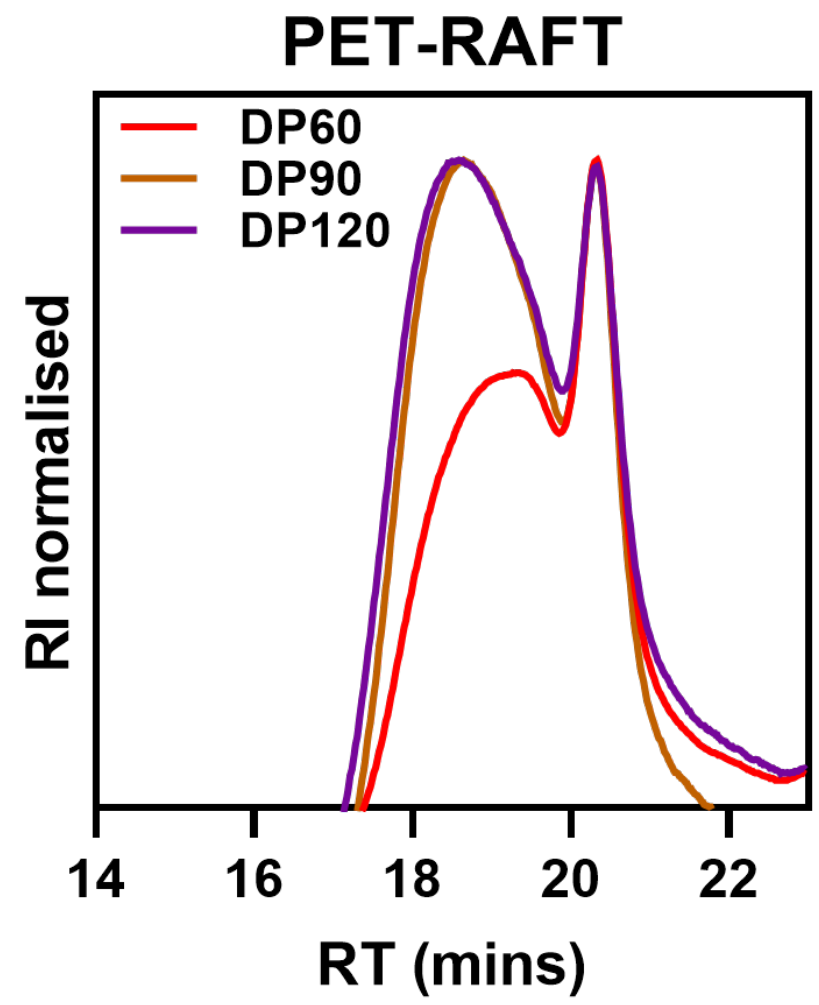

Figure S3: GPC traces of PEG_{113}-$PDMAEMA_{60,90,120}$ block copolymers (synthesised via PET-RAFT) in DMAc.

Table S2. GPC Characterisation Results of PEG_{113}-$PDMAEMA_n$ polymers synthesised via PET-RAFT polymerisation.

Polymer	$M_{ntheo}{}^a$	M_{nGPC}	Đ
PEG_{113}-$PDMAEMA_{60}$	14,800	19,800	1.51
PEG_{113}-$PDMAEMA_{90}$	18,500	20,500	1.75
PEG_{113}-$PDMAEMA_{120}$	25,200	21,200	1.76

[a] Mn_{theo} was calculated by adding the known $M_{wPEG113}$ to the theoretical $M_{wDMAEMA}$ multiplied by the DP calculated by ^1H NMR.

Table S3. qPCR results showing the levels of DNA amplifiability relative to the control after PET-RAFT or UV treatment.

Sample	Mean C_t	ΔC_t	$2^{-\Delta C_t}$	Relative amplifiable DNA
DNA	4.98	–	1.00	100%
PET-RAFT 1	4.51	-0.47	1.38	100%
PET-RAFT 2	4.67	-0.318	1.25	100%
PET-RAFT 3	5.02	0.033	0.98	98%
UV 10min	7.12	2.14	0.23	23%
UV 1h	16.40	11.42	0.0004	0.04%

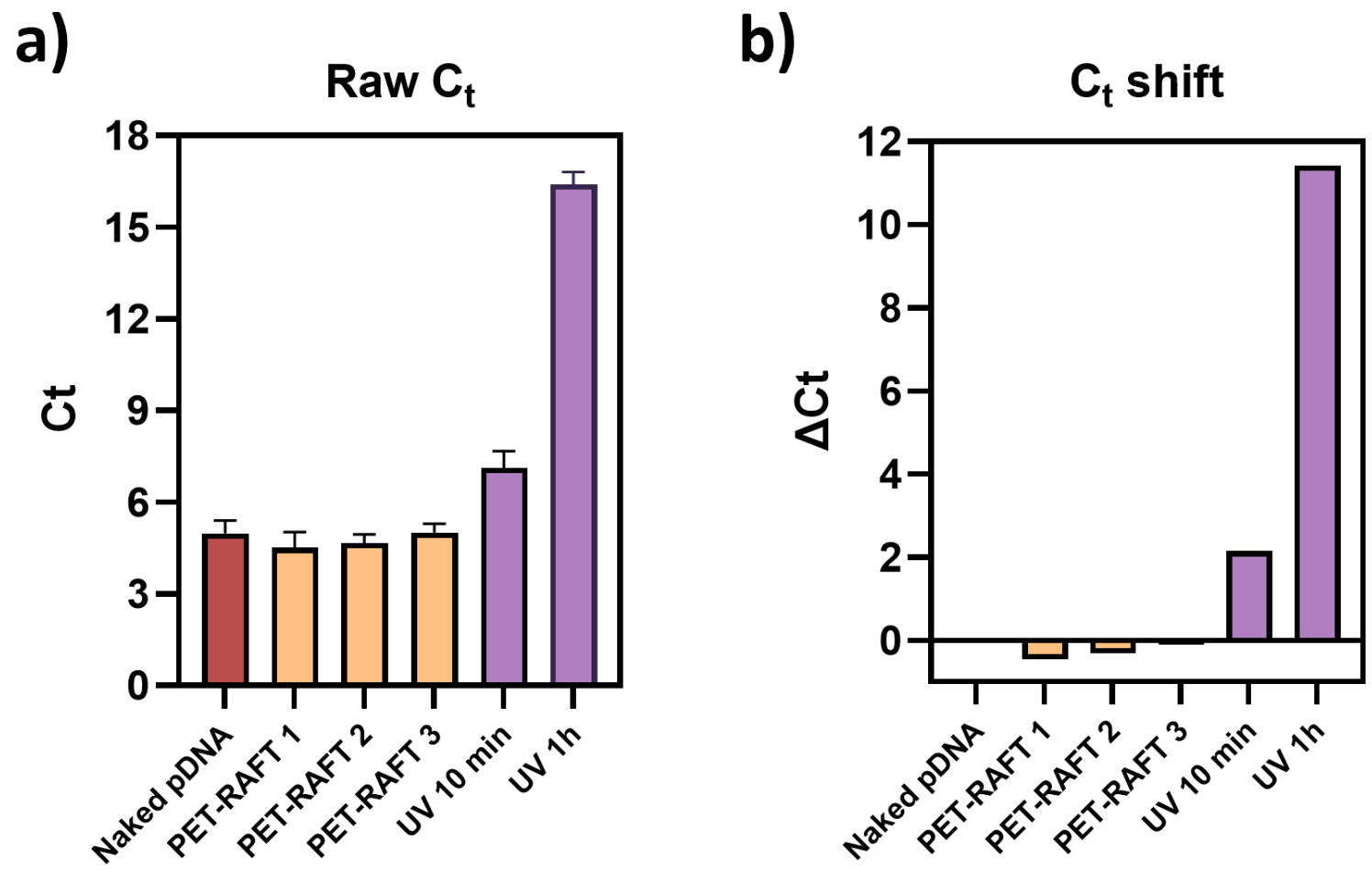

Figure S4: Quantitative PCR (qPCR) analysis of DNA integrity following PET-RAFT and UV treatment. a) Raw ΔC_t values obtained from each sample. b) ΔC_t values relative to the untreated control, where higher ΔC_t indicates reduced amplifiable DNA.

Table S4. Zeta potential of polyplexes in 0.1 mM KCl (pH$\sim$ 6.5).

Assembly method	DP_{DMAEMA}	N/P ratio	Zeta potential (mV)[a]
Conventional	60	10	32.3 ± 2.84
Conventional	60	20	34.5 ± 0.39
Conventional	60	30	31.2 ± 0.27
Conventional	90	10	27.5 ± 0.96
Conventional	90	20	27.7 ± 1.18
Conventional	90	30	31.5 ±0.53
Conventional	120	10	37.6 ± 0.35
Conventional	120	20	18.0 ± 0.11
Conventional	120	30	30.6 ± 0.26
PIESA	60	10	-20.8 ± 0.57
PIESA	60	20	-0.1 ± 0.26
PIESA	60	30	1.3 ± 0.10
PIESA	90	10	-2.7 ± 0.94
PIESA	90	20	-5.6 ± 1.27
PIESA	90	30	-0.4 ± 0.01
PIESA	120	10	-17.3 ± 0.46
PIESA	120	20	2.7 ± 0.11
PIESA	120	30	5.4 ± 0.52

[a] Average of 3 repeats.

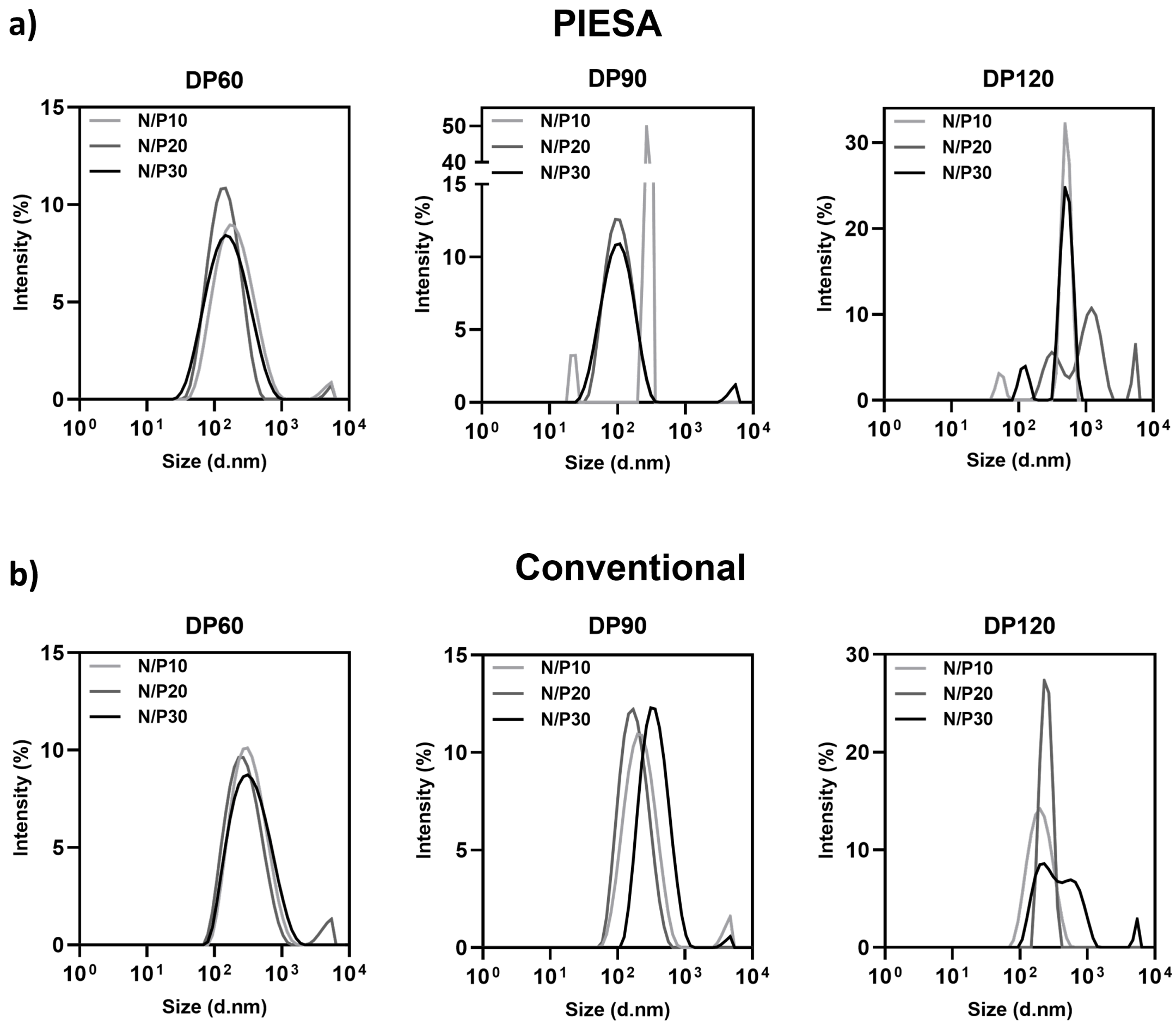

Figure S5: DLS intensity traces showing the hydrodynamic diameter distributions of PIESA (a) and Conventional (b) polyplexes across different formulations. Each trace corresponds to the average of three independent measurements per formulation.

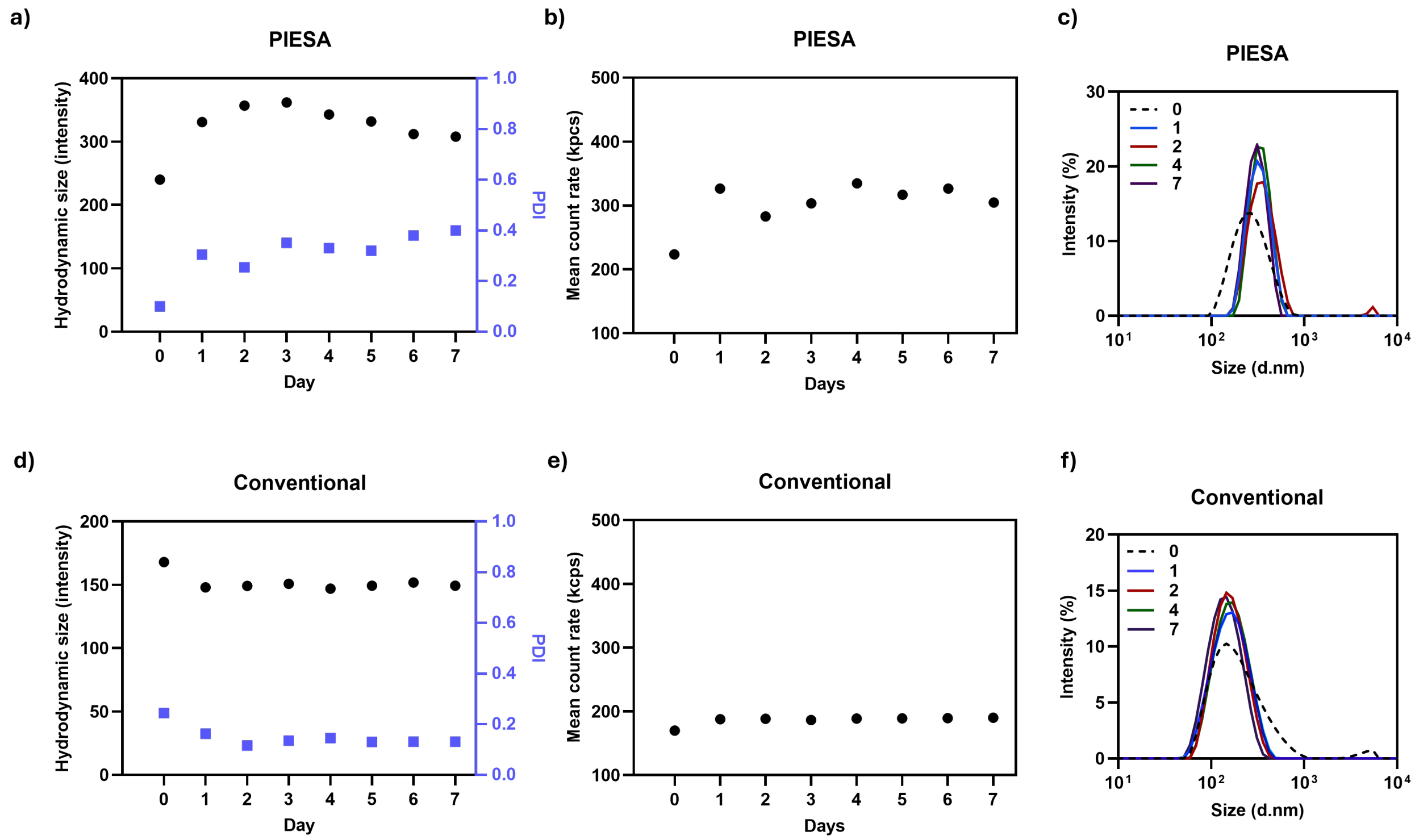

Figure S6: DLS measurements of PIESA-derived (a–c) and conventionally assembled (d–f) polyplexes at DP 90 N/P = 10. Polyplexes were diluted to 0.05 w/w % in deionised water, filtered (nylon, 0.2 μm) and analysed daily over 7 days at room temperature for hydrodynamic diameter, polydispersity index (PDI) and mean count rate (kcps). Samples were gently vortexed before each measurement. Each trace represents the average of three independent measurements per formulation. For clarity, only 5 time points are shown in the traces (c and f).

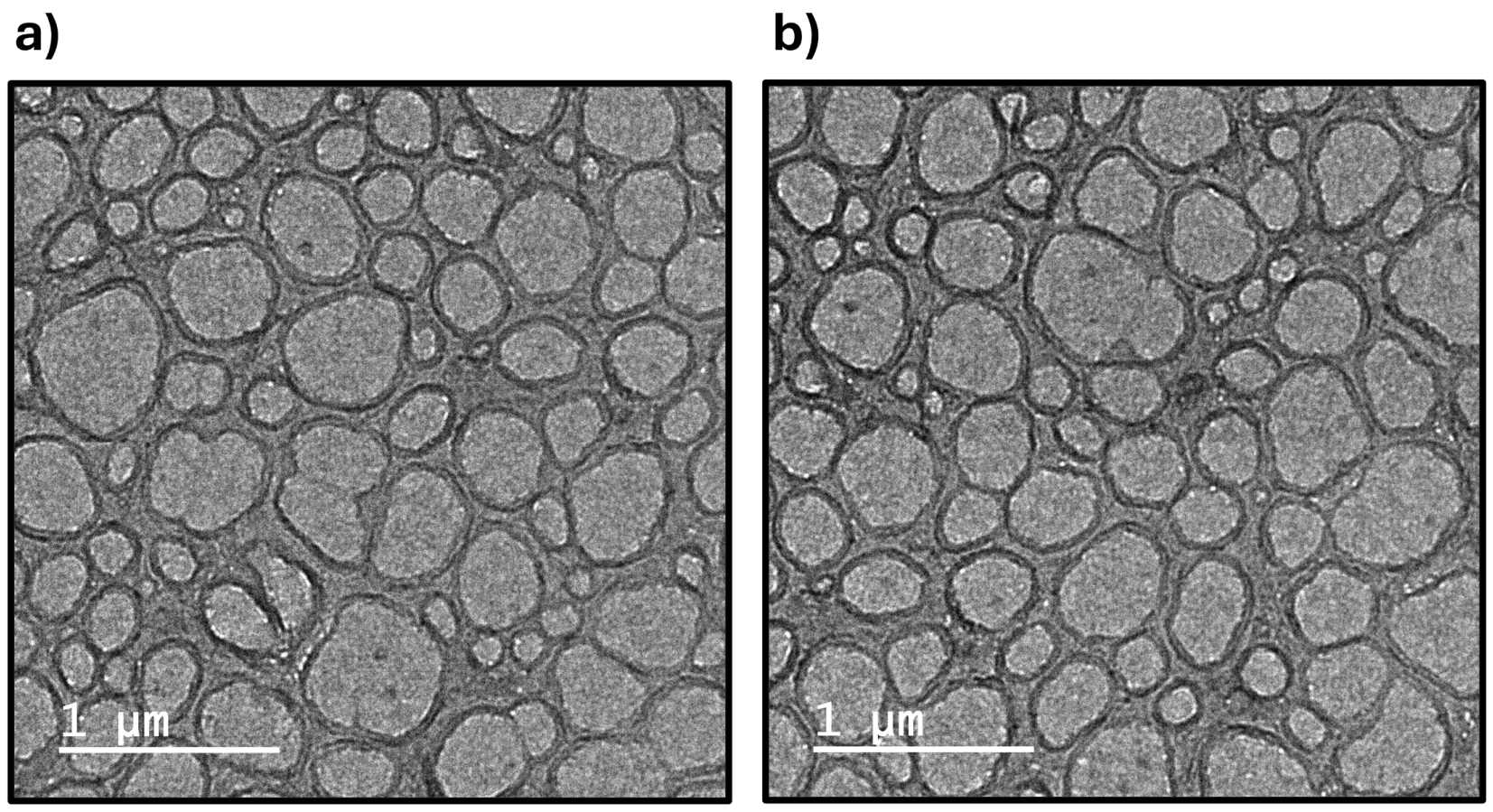

Figure S7: TEM images of PEG_{113}-$PDMAEMA_{83}$ polyplexes conventionally assembled at N/P10. Nanoparticles exhibit quasi-spherical and irregular contours. The samples were dried overnight on the carbon-coated copper grids and the images were captured using uranyl acetate staining. a-b) Scale bar = 1 μm.

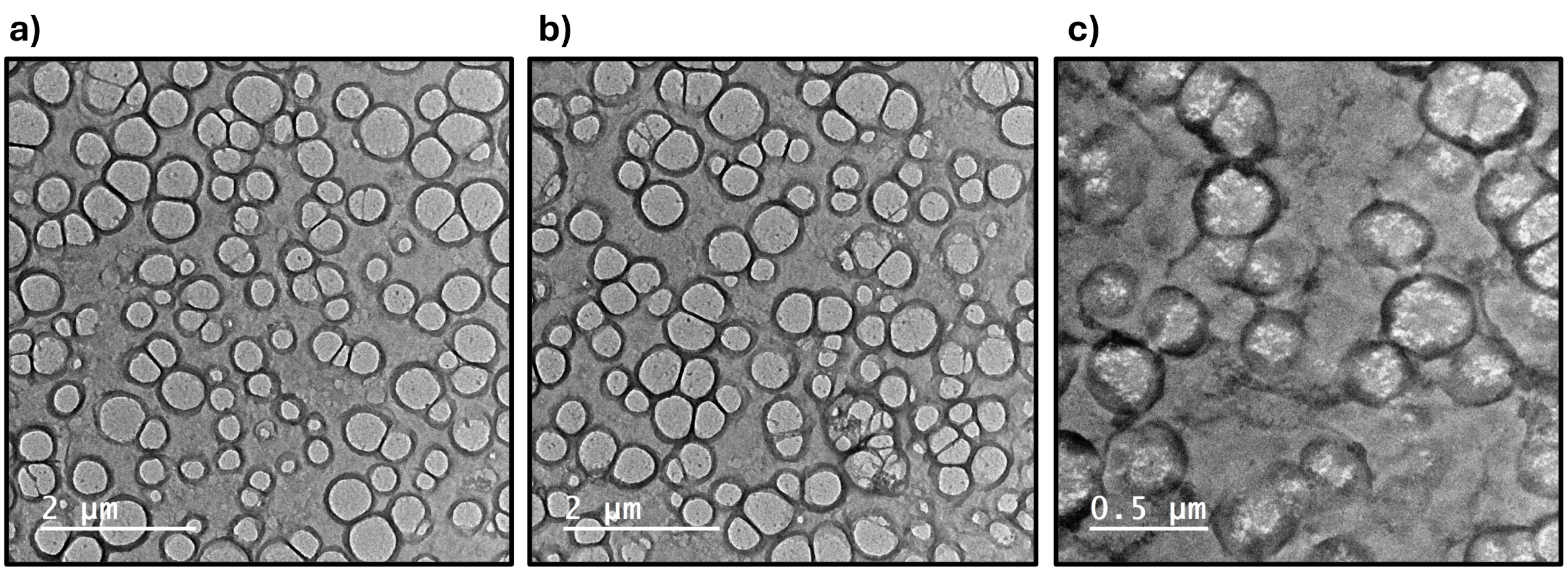

Figure S8: TEM images of PEG_{113}-$PDMAEMA_{90}$ PIESA polyplexes assembled at N/P10. Nanoparticles exhibit quasi-spherical and regular contours. The samples were dried overnight on the carbon-coated copper grids and the images were captured using uranyl acetate staining. a-b) Scale bar = 2 μm and c) 0.5 μm.

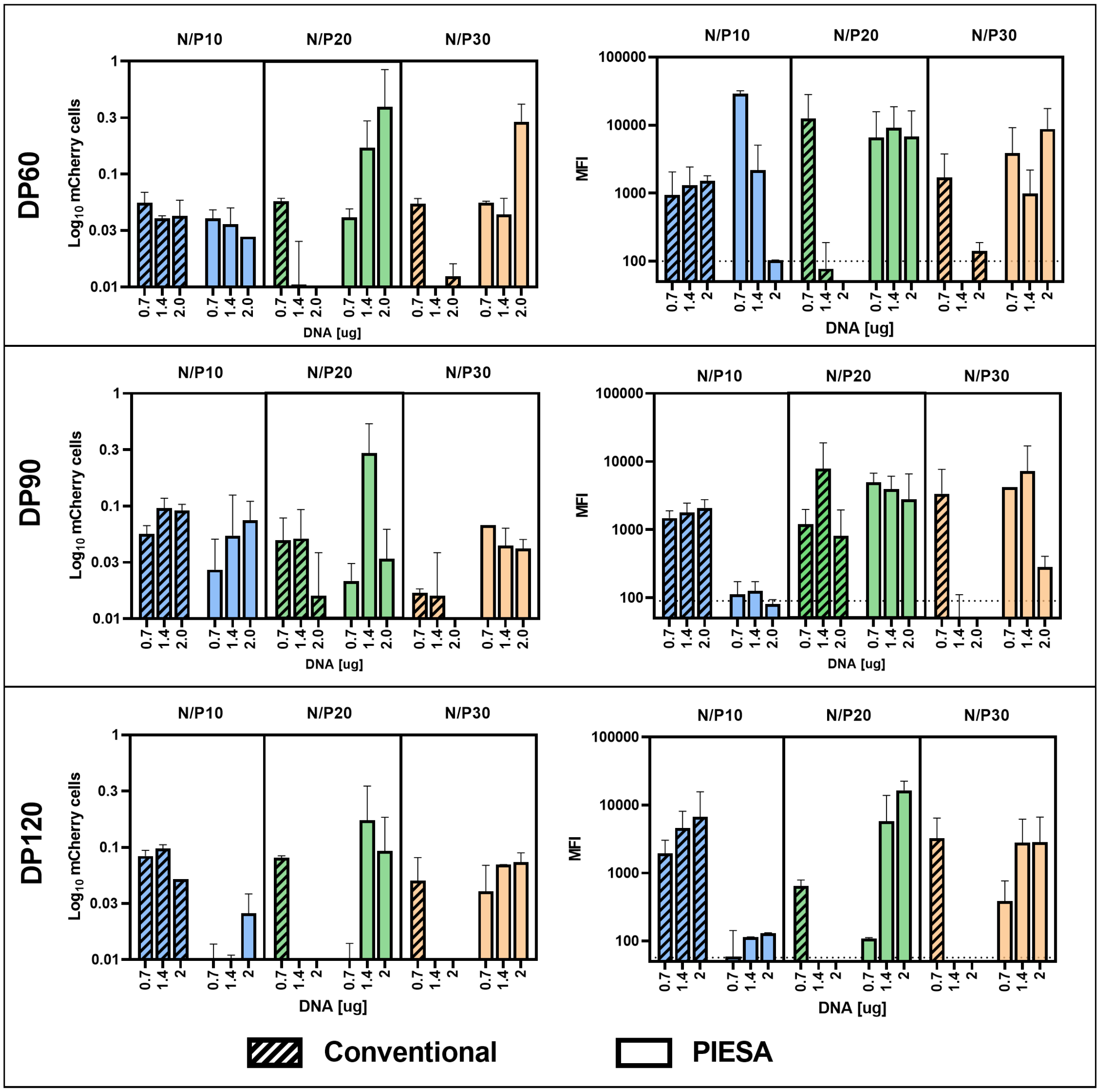

Figure S9: Flow cytometry analysis of cells transfected with conventional polyplexes (diagonal striped bars) and PIESA polyplexes (unfilled bars). The relative transfection efficiency is presented on the Y-axis as a bar chart. The bar charts on the right represent the Mean Fluorescent Intensity (MFI) of cells expressing mCherry. The comparison illustrates the efficiency of conventional versus PIESA polyplexes across different pDNA dosages and polyplex formulation.

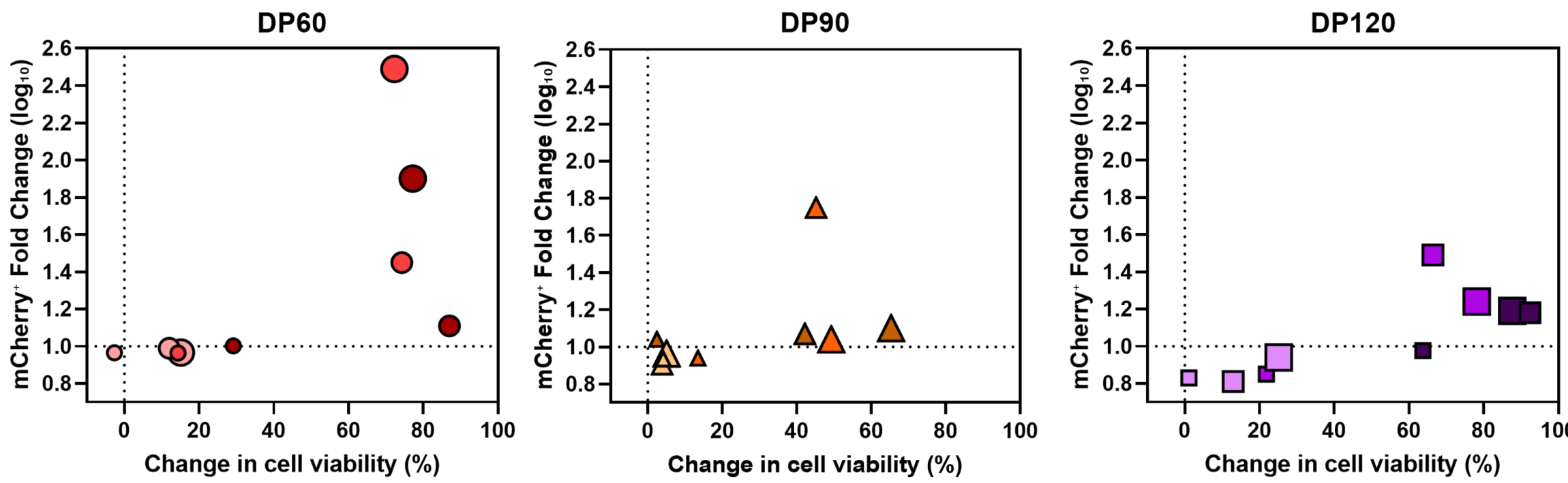

Figure S10: Scatter plot graphs show the flow cytometry analysis of the transfection efficiency in relation to the cytotoxicity each polyplex formulation. Each symbol represents an individual formulation where the shape indicates the polymer DP (circles, triangles or squares), the shade reflects the N/P ratio (darker color indicates a higher N/P ratio), and the symbol size corresponds to the DNA dose carried (0.7, 1.4, or 2μg per well). The Y-axis shows the mCherry + cells fold change for each sample (Conventional vs PIESA) calculated as $10^{(log10\, mCherry\, cells\, Conventional - PIESA)}$; The dashed line at Fold = 1 indicates no change between conditions; points above the line mean PIESA is greater; points below mean it is lower.

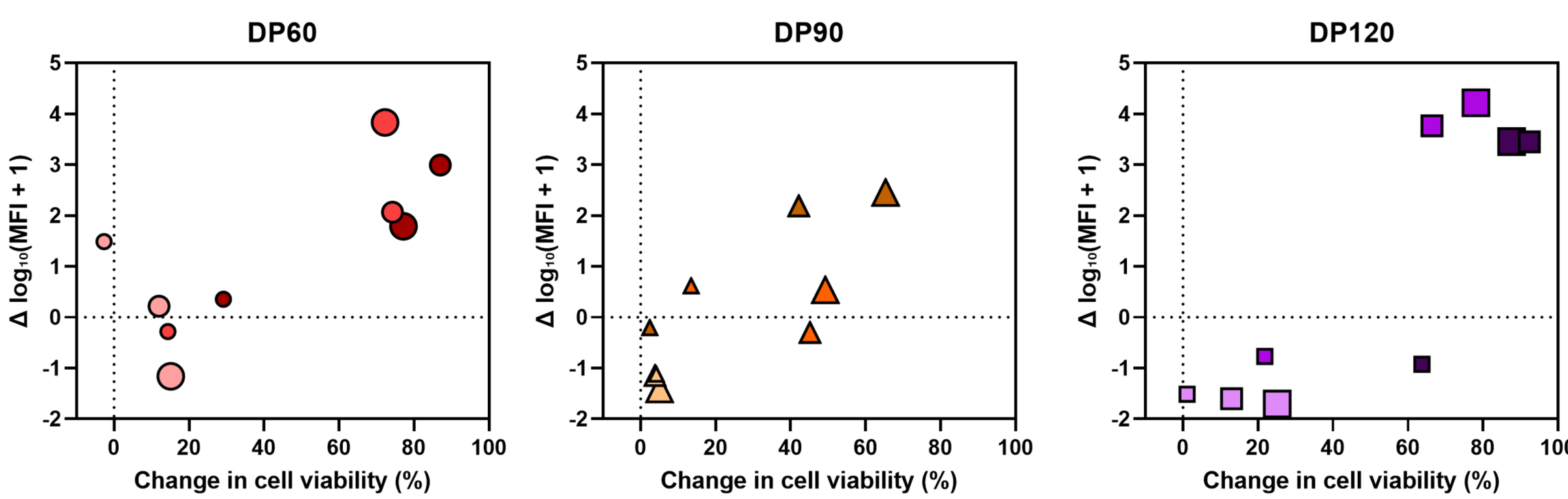

Figure S11: Scatter plot graphs show the flow cytometry analysis of the transfection efficiency in relation to the cytotoxicity each polyplex formulation. Each symbol represents an individual formulation where the shape indicates the polymer DP (circles, triangles or squares), the shade reflects the N/P ratio (darker color indicates a higher N/P ratio), and the symbol size corresponds to the DNA dose carried (0.7, 1.4, or 2μg per well). The Y-axis shows MFI log difference values represented as $\Delta \log_{10} = \log_{10}(MFI_{PIESA} + 1) - \log_{10}(MFI_{Conventional} + 1)$ to avoid zeros. The dashed line at Fold = 1 shows no change. Higher values show strong increases in fluorescence signal in PIESA.

References

(1) Wan, J.; Fan, B.; Thang, S. H. Sonochemical preparation of polymer–metal nanocomposites with catalytic and plasmonic properties. *Nanoscale Advances* **2021**, *3*, 3306–3315.
